# Comprehensive pharmacovigilance assessment of pairwise drug–drug interactions and acute pancreatitis in the FDA adverse event reporting system: focus on incretin-based drugs

**DOI:** 10.3389/fdsfr.2026.1827098

**Published:** 2026-06-10

**Authors:** Issei Kazume, Kenji Onda, Sachiko Tanaka, Takashi Yokokawa, Kenichi Suzuki

**Affiliations:** 1 Department of Clinical Pharmacology, School of Pharmacy, Tokyo University of Pharmacy and Life Sciences, Tokyo, Japan; 2 P-One Clinic, Keikokai Medical Corp, Tokyo, Japan

**Keywords:** acute pancreatitis, DPP-4 inhibitors, drug–drug interaction, FAERS, GLP-1 receptor agonists

## Abstract

**Background:**

Drug-induced pancreatitis remains a clinically important condition owing to its severity and relevance to pharmacotherapeutic risk management. Incretin-based antidiabetic agents, including glucagon-like peptide-1 receptor agonists (GLP-1 RAs) and dipeptidyl peptidase-4 (DPP-4) inhibitors, are associated with an increased risk of acute pancreatitis (AP). However, the extent to which concomitant medication modifies AP has not yet been systematically elucidated. We conducted a comprehensive systematic drug–drug interaction (DDI) assessment involving GLP-1 RA and DPP-4 inhibitors using the U.S. Food and Drug Administration’s Adverse Event Reporting System (FAERS).

**Methods:**

Disproportionality analyses were conducted using the FAERS data (JAPIC FAERS) from the fourth quarter of 1997 to the first quarter of 2024. Crude reporting odds ratios (cRORs) were calculated to assess the association between individual drugs and AP. DDI signals for 1:1 concomitant use involving GLP-1 RAs or DPP-4 inhibitors were comprehensively evaluated using the Ω shrinkage measure, with Ω_025_ as the signal detection criterion. Ω_025_ were analyzed for all pooled cases, and temporal trends were assessed across six multi-year periods using a period-stratified analysis.

**Results:**

Elevated cRORs for AP were observed for several agents, including GLP-1 RAs, DPP-4 inhibitors, and other antidiabetic medications. Pairwise DDI analyses of GLP-1 RAs or DPP-4 inhibitors with other drugs identified positive interaction signals in 308 drug pairs in pooled analyses. Period-stratified analyses narrowed the findings to 11 drug pairs with consistent signals across ≥3 of 6 periods, including dulaglutide–atorvastatin (four periods). However, no concomitant drug combinations involving GLP-1 RAs or DPP-4 inhibitors were identified as being robustly associated with increased AP reporting.

**Conclusion:**

Period-stratified analysis helped mitigate false-positive signals identified in the pooled DDI analyses. Drug pairs exhibiting recurrent weak AP signals warrant continued pharmacovigilance and further evaluation.

## 1 Introduction

Acute pancreatitis (AP) is a leading gastrointestinal disorder that requires hospitalization and is a potentially fatal condition (Boxhoorn et al., 2020; Iannuzzi et al., 2022). The global annual incidence is estimated at 34 cases per 100,000, and the associated healthcare costs are reported to reach approximately USD 9.3 billion per year. Notably, the incidence of AP continues to increase worldwide (Xiao et al., 2016; Iannuzzi et al., 2022; Subramanian et al., 2024), with its etiologies including gallstones, alcohol consumption, hypertriglyceridemia, drug-induced causes, and infections. Among these, drug-induced pancreatitis accounts for approximately 2.8%–5.3% of all cases; although relatively uncommon, it necessitates careful consideration in pharmacotherapy. To date, several classes of medications, such as antidiabetic agents, anticancer drugs, and antiviral agents, have been reported to have established or suspected causal associations with AP, based on adverse event and case reports (Saini et al., 2023; Li et al., 2024).

Glucagon-like peptide-1 receptor agonists (GLP-1 RAs) and dipeptidyl peptidase-4 (DPP-4) inhibitors are antidiabetic agents that target incretin hormones, and currently play a central role in the treatment of type 2 diabetes mellitus (Drucker, 2006; Neumiller, 2015). In addition to their glucose-lowering effects, GLP-1 RAs promote weight loss and have been increasingly used for the treatment of obesity in recent years, with further expansion of their anticipated use (Drucker, 2022). However, these agents also include warnings regarding the development of AP in their package insert, and their association with AP remains a subject of ongoing debate (Pratley et al., 2024).

Although previous studies have examined the relationship between individual agents and the occurrence of pancreatitis (Zhang et al., 2022; Nreu et al., 2023; Alenzi et al., 2024; Tuersun et al., 2024), information regarding the extent to which the risk of AP is influenced when these drugs are used concomitantly with other medications remains limited. When multiple drugs are administered concurrently, drug–drug interactions (DDI) may increase the risk of AP; therefore, elucidating such interactions and appropriately assessing and mitigating the associated risks are essential for ensuring the safety of pharmacotherapy. Nevertheless, existing DDI research on drug-induced pancreatitis is scarce, and evaluations of the pancreatitis risk under polypharmacy conditions remain insufficient.

The U.S. Food and Drug Administration’s Adverse Event Reporting System (FAERS) is a spontaneous reporting database that collects information on adverse events associated with the use of a wide range of therapeutic agents (Sakaeda et al., 2013). Large-scale databases such as FAERS are particularly valuable for analyzing rare adverse events. Moreover, FAERS is an important resource for detecting potential DDIs and identifying previously unrecognized adverse events (Tatonetti et al., 2012); its application has been proposed for evaluating DDIs and identifying medications that may mitigate adverse drug reactions (Zhao et al., 2013).

Disproportionality analysis is commonly used to detect signals of drug–adverse event associations. Given the inherent limitations of spontaneous reporting data, such as the inability to estimate the true incidence rates of adverse drug reactions and the presence of reporting biases, direct comparisons of reporting odds ratios (RORs) derived from disproportionality analyses are considered inappropriate. However, multivariate analyses that incorporate logistic regression have been proposed to enable comparisons using adjusted RORs under specific conditions (Oshima et al., 2018; Zamami et al., 2019; Onda et al., 2023). Furthermore, several methods have been developed to detect DDI signals between two agents, including the Ω shrinkage measure model, additive and multiplicative models, and the combination risk ratio model (Noguchi et al., 2019). Although no standardized approach has been established, the Ω shrinkage measure model has been reported as the most conservative among these methods (Noguchi et al., 2020).

In the current study, we conducted a 1:1 pairwise DDI analysis of AP reports involving GLP-1 RAs or DPP-4 inhibitors in combination with other medications, using FAERS. Through this approach, we aimed to identify the relevant DDIs associated with AP reporting.

## 2 Materials and methods

### 2.1 Data source and mining

This analysis was conducted using data from the Japan Pharmaceutical Information Center FAERS (JAPIC FAERS), covering the fourth quarter of 1997 to the first quarter of 2024. JAPIC FAERS is a database preprocessed by the Japan Pharmaceutical Information Center based on the original US FAERS data and has undergone data cleaning to ensure quality, including the removal of duplicate reports pertaining to the same patient. Because the FAERS is a publicly accessible and anonymized database, institutional review board approval was deemed unnecessary. This study followed the READUS-PV (REporting of A Disproportionality analysis for druUg Safety signal detection using individual case safety reports in PharmacoVigilance) guidelines (Fusaroli et al., 2024). The FAERS database consists of seven distinct tables, of which the drug (DRUG) and adverse event (REAC) tables were used. These tables were connected through PrimaryID and analyzed using a relational database software (Microsoft Access for Microsoft 365). We confirmed the validity of our aggregation system by demonstrating consistency with results reported in previous studies (Alkabbani et al., 2022; Noguchi, 2022; Honma et al., 2024).

In the DRUG table, each medication is classified according to its involvement in adverse events as a Primary Suspected (PS) drug, Secondary Suspected (SS) drug, concomitant (C) drug, or an interacting (I) drug. In the present study, all drug classifications were included in both the disproportionality analysis for individual drugs (for the calculation of ROR) and the DDI analysis. Medications registered under the same PrimaryID were defined as concomitant drugs and analyzed accordingly. In the DDI analysis, we regarded secondary suspected and interacting drugs as equivalent to concomitant drugs and did not apply a hierarchical classification based on drug role.

### 2.2 Definition of adverse events

All event reports were identified using the Preferred Terms (PTs) outlined in the Medical Dictionary for Regulatory Activities (MedDRA; version 27.0). AP was defined based on the PTs included in the Standard MedDRA Queries 20000022 with a narrow scope. A complete list of the PTs is provided in the Supplementary Table S1.

### 2.3 Disproportionality analysis of acute pancreatitis reports

**FIGURE 1:**
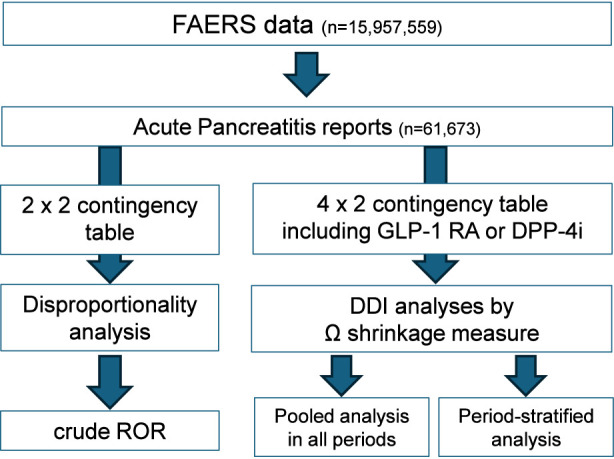
Flow chart for determining crude reporting odds ratios (cRORs) and assessing drug–drug interactions (DDIs) in acute pancreatitis (AP) reports. A disproportionality analysis focusing on individual drugs associated with AP was conducted using 2 × 2 contingency tables to calculate cRORs. In addition, pair-wise DDI analyses were performed using the Ω shrinkage measure based on 4 × 2 contingency tables. Both pooled analyses covering the entire 26.5-year study period and period-stratified analyses were conducted.

**FIGURE 2:**
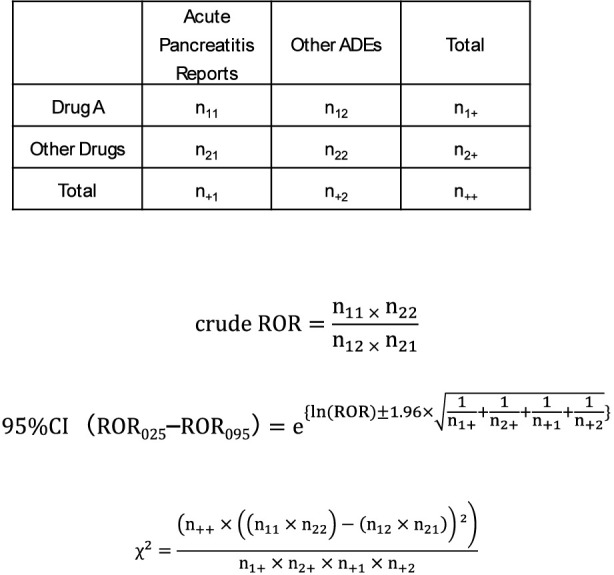
The cROR for Drug A and AP was calculated using the upper 2 × 2 contingency table among all reported cases.

The overall analysis was conducted in accordance with the flowchart shown in Figure 1. First, a disproportionality analysis of the drugs associated with AP was performed. A 2 × 2 contingency table was constructed based on the number of reports on drug exposure and AP for all drugs registered in FAERS (8,585 distinct agents). Crude reporting odds ratios, 95% confidence intervals, and chi-squared values were subsequently calculated (Figure 2).

### 2.4 Pairwise drug–drug interaction analysis of acute pancreatitis reporting involving GLP-1 receptor agonists or DPP-4 inhibitors

**FIGURE 3:**
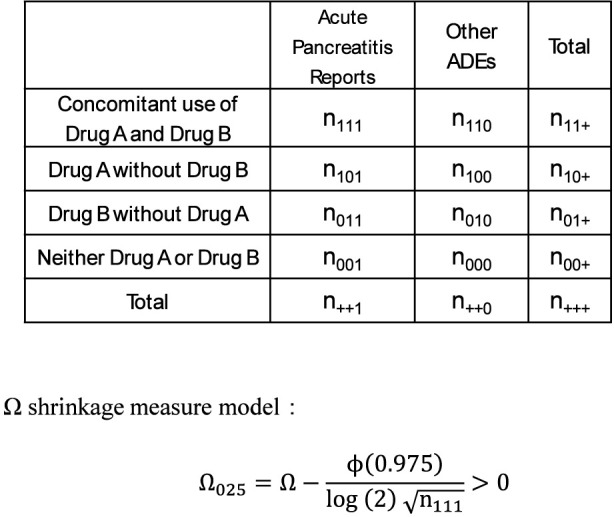
DDI signal analyses were performed using 4 × 2 contingency tables constructed from the number of reported AP cases. Interaction signals were evaluated using the Ω shrinkage measure model. Drug A was defined as glucagon-like peptide-1 receptor agonists (GLP-1 RAs) or dipeptidyl peptidase-4 (DPP-4) inhibitors, and Drug B was defined as all other concomitant medications (8,584 distinct agents).

A DDI analysis was performed using the Ω shrinkage measure, a validated method for interaction signal detection in spontaneous reporting databases (Norén et al., 2008; Honma et al., 2024). We comprehensively evaluated the interaction signals of one-to-one combinations of glucagon-like peptide-1 RAs (seven agents: albiglutide, dulaglutide, exenatide, liraglutide, lixisenatide, semaglutide, and tirzepatide) and dipeptidyl peptidase-4 inhibitors (alogliptin, linagliptin, saxagliptin, sitagliptin, and vildagliptin) with all the other reported medications (8,584 agents). The interaction signal metric (Ω_025_) for each drug pair was calculated and summarized, as illustrated in Figure 3. To assess the temporal consistency of the detected interaction signals, the study period (26.5 years) was divided into six consecutive intervals of several years. Ω_025_ values were calculated separately for each interval, and temporal trends in interaction signal strength were evaluated. For the period-stratified analysis, we used six time periods derived from the predefined twelve periods in the JAPIC FAERS dataset by combining adjacent intervals to ensure sufficient sample size and stability of the estimates. The periods were defined as follows: Period 1, 1997 Q4–2013 Q4; Period 2, 2014 Q1–2017 Q2; Period 3, 2017 Q3–2019 Q2; Period 4, 2019 Q3–2021 Q1; Period 5, 2021 Q2–2022 Q3; and Period 6, 2022 Q4–2024 Q1.

## 3 Results

### 3.1 Disproportionality analysis of AP reports

The cleaned FAERS dataset comprised 15,957,559 reports, of which 61,673 were identified as AP cases. Among the evaluated drugs, 1,247 were associated with significantly elevated crude RORs (cRORs). Table 1 presents the top 30 drugs ranked by χ^2^ values, as identified through the disproportionality analysis for AP. The highest reporting signal was observed for liraglutide, with a cROR (95% CI) of 19.86 (19.14–20.60). Elevated cRORs were also identified for sitagliptin at 10.90 (10.51–11.30), metformin at 4.50 (4.39–4.62), exenatide at 8.99 (8.61–9.39), and eluxadoline at 40.23 (36.00–44.96). When stratified by pharmacological class, a substantial number of drugs with increased cRORs were associated with glucose-lowering therapy, including GLP-1 RAs (liraglutide, exenatide, dulaglutide, and semaglutide), DPP-4 inhibitors (sitagliptin, linagliptin, and saxagliptin), metformin, glimepiride, and insulin. In addition to antidiabetic agents, elevated cRORs were observed for eluxadoline, which is indicated for irritable bowel syndrome; fenofibrate, which is prescribed for hypertriglyceridemia; and several agents used in oncology, human immunodeficiency virus treatment, and antipsychotic therapy.

**TABLE 1:** Disproportionality analysis of acute pancreatitis (AP) reports.

Drug	n_11_	n_12_	n_21_	n_22_	cROR	Χ^2^
Liraglutide	3,235	44,193	58,438	15,851,693	19.86 (19.14–20.6)	51155.8
Sitagliptin	3,132	77,662	58,541	15,818,224	10.9 (10.51–11.3)	25692
Metformin	7,015	440,422	54,658	15,455,464	4.5 (4.39–4.62)	16687.2
Exenatide	2,208	65,354	59,465	15,830,532	8.99 (8.61–9.39)	14634.4
Eluxadoline	361	2,326	61,312	15,893,560	40.23 (36–44.96)	11885.6
Fenofibrate	1,391	49,080	60,282	15,846,806	7.45 (7.06–7.86)	7384.23
Dulaglutide	1,707	72,447	59,966	15,823,439	6.22 (5.92–6.53)	7100.16
Semaglutide	1,101	35,406	60,572	15,860,480	8.14 (7.66–8.65)	6570.98
Pegaspargase	526	9,142	61,147	15,886,744	14.95 (13.69–16.33)	6418.72
Didanosine	456	6,993	61,217	15,888,893	16.92 (15.39–18.61)	6367.12
Quetiapine	2,363	144,806	59,310	15,751,080	4.33 (4.16–4.52)	5734.71
Olanzapine	1,586	85,911	60,087	15,809,975	4.86 (4.62–5.11)	4648
Asparaginase	425	8,973	61,248	15,886,913	12.29 (11.14–13.55)	4177.87
Azathioprine	1,361	77,997	60,312	15,817,889	4.58 (4.34–4.83)	3656.39
Pancrelipase	653	23,341	61,020	15,872,545	7.28 (6.73–7.87)	3403.26
Linagliptin	605	22,530	61,068	15,873,356	6.98 (6.44–7.57)	2988.95
Stavudine	348	8,557	61,325	15,887,329	10.54 (9.46–11.73)	2869.91
Mesalazine	1,141	71,315	60,532	15,824,571	4.18 (3.94–4.44)	2669.51
Mercaptopurine	460	15,522	61,213	15,880,364	7.69 (7–8.44)	2580.06
Glimepiride	991	58,473	60,682	15,837,413	4.42 (4.15–4.71)	2540.38
Hydrochlorothiazide	2,948	312,157	58,725	15,583,729	2.51 (2.41–2.6)	2517.33
Pantoprazole	2,966	320,292	58,707	15,575,594	2.46 (2.37–2.55)	2416.93
Insulin	3,743	451,738	57,930	15,444,148	2.21 (2.14–2.28)	2307.55
Lisinopril	2,756	298,197	58,917	15,597,689	2.45 (2.35–2.54)	2231.95
Saxagliptin	301	8,299	61,372	15,887,587	9.39 (8.37–10.54)	2166.65
Ezetimibe	1,080	80,696	60,593	15,815,190	3.49 (3.29–3.71)	1863.33
Simvastatin	2,400	265,362	59,273	15,630,524	2.39 (2.29–2.48)	1838.71
Pancreatin	306	9,845	61,367	15,886,041	8.05 (7.18–9.02)	1822.17
Rufloxacin	11	7	61,662	15,895,879	405.1 (157.03–1045.04)	1724.07
Tigecycline	166	3,341	61,507	15,892,545	12.84 (10.98–15.01)	1721.65

n_11_, number of reports including both the target drug and acute pancreatitis; n_12_, reports including the target drug without acute pancreatitis; n_21_, reports excluding the target drug but including acute pancreatitis; n_22_, reports excluding both the target drug and acute pancreatitis; cROR, crude reporting odds ratio; χ^2^, chi-square value calculated from the corresponding 2 × 2 contingency table.

### 3.2 Pairwise DDI analysis for AP reporting involving GLP-1 RAs or DPP-4 inhibitors

A comprehensive DDI analysis was conducted using data registered in the FAERS database to evaluate the impact of one-to-one drug combinations on AP reporting. Using the Ω shrinkage measure model described in the Materials and Methods section, interaction signal estimates (Ω_025_) were systematically calculated for pairwise combinations of GLP-1 RAs or DPP-4 inhibitors with all other reported medications. In the analysis encompassing the entire study period from 1997 to 2024, positive interaction signals based on Ω_025_ values were identified for 308 drug pairs, corresponding to 0.3% of all evaluated combinations (103,008 pairs). The most frequently reported drug pair was insulin in combination with exenatide, with 532 reports exhibiting a Ω_025_ value of 0.04. This was followed by acetylsalicylate in combination with exenatide, which accounted for 436 reports and exhibited an Ω_025_ value of 1.1. Other highly-ranked combinations included exenatide with lisinopril (n_111_ = 381, Ω_025_ = 1.15), acetylsalicylate with liraglutide (n_111_ = 347, Ω_025_ = 0.03), exenatide with simvastatin (n_111_ = 331, Ω_025_ = 1.42), exenatide with hydrochlorothiazide (n_111_ = 329, Ω_025_ = 1.28), and atorvastatin with exenatide (n_111_ = 300, Ω_025_ = 0.84) (Table 2).

**TABLE 2:** Drug–drug interaction analysis of AP between incretin-based drugs and concomitant medications in all pooled periods.

Drug A	Drug B	n_111_	n_11+_	E_111_	Ω (Ω_025_−Ω_975_)	Judge
Exenatide	Insulin	532	13,773	476.4	0.16 (0.04–0.28)	P
Acetylsalicylate	Exenatide	463	6,536	196.99	1.23 (1.1–1.36)	P
Exenatide	Lisinopril	381	4,661	154.66	1.3 (1.15–1.44)	P
Acetylsalicylate	Liraglutide	347	4,437	305.22	0.18 (0.03–0.34)	P
Exenatide	Simvastatin	331	3,342	110.85	1.57 (1.42–1.73)	P
Exenatide	Hydrochlorothiazide	329	3,575	121.13	1.44 (1.28–1.59)	P
Atorvastatin	Exenatide	300	4,469	149.2	1.01 (0.84–1.17)	P
Liraglutide	Lisinopril	273	2,488	174.34	0.65 (0.47–0.82)	P
Hydrochlorothiazide	Liraglutide	247	2,434	172.47	0.52 (0.34–0.7)	P
Exenatide	Paracetamol	236	2,159	69.67	1.75 (1.57–1.94)	P
Liraglutide	Simvastatin	213	2,000	141.18	0.59 (0.4–0.79)	P
Exenatide	Fenofibrate	199	1,438	71.8	1.46 (1.26–1.66)	P
Exenatide	Metoprolol	187	2,919	95.95	0.96 (0.75–1.17)	P
Exenatide	Furosemide	181	2,603	85.67	1.07 (0.86–1.28)	P
Liraglutide	Sitagliptin	170	1,466	141.37	0.27 (0.05–0.48)	P
Exenatide	Levothyroxine	169	3,527	117.5	0.52 (0.3–0.74)	P
Exenatide	Ezetimibe	168	1,630	62.27	1.42 (1.21–1.64)	P
Glimepiride	Liraglutide	165	1,751	134.08	0.3 (0.08–0.52)	P
Amlodipine	Exenatide	163	2,646	89.25	0.87 (0.64–1.09)	P
Exenatide	Omeprazole	149	2,344	81.31	0.87 (0.64–1.1)	P
Exenatide	Rosuvastatin	137	1,935	67.9	1.01 (0.77–1.25)	P
Atorvastatin	Dulaglutide	133	3,226	82.97	0.68 (0.43–0.92)	P
Liraglutide	Pioglitazone	121	1,203	90.66	0.41 (0.16–0.67)	P
Clopidogrel	Exenatide	117	1,520	49.42	1.23 (0.97–1.5)	P
Exenatide	Valsartan	116	1,553	49.6	1.22 (0.95–1.48)	P
Esomeprazole	Exenatide	113	1,617	58.18	0.95 (0.69–1.22)	P
Exenatide	Pantoprazole	106	1,228	45	1.23 (0.95–1.5)	P
Acetylsalicylate	Dulaglutide	102	2,936	71.98	0.5 (0.22–0.78)	P
Exenatide	Ramipril	100	1,024	37.68	1.4 (1.11–1.68)	P
Atenolol	Exenatide	100	1,141	39.73	1.32 (1.04–1.6)	P

n_111_, number of reports including Drug A, Drug B, and AP; n_11+_, reports including both drugs; E_111_, expected value of the Ω shrinkage measure model; P, positive signal. Drug pairs with n_111_ ≥ 100 were listed. Drug pairs (Drugs A and B) are presented in alphabetical order.

### 3.3 Temporal analysis of interaction signals

**FIGURE 4:**
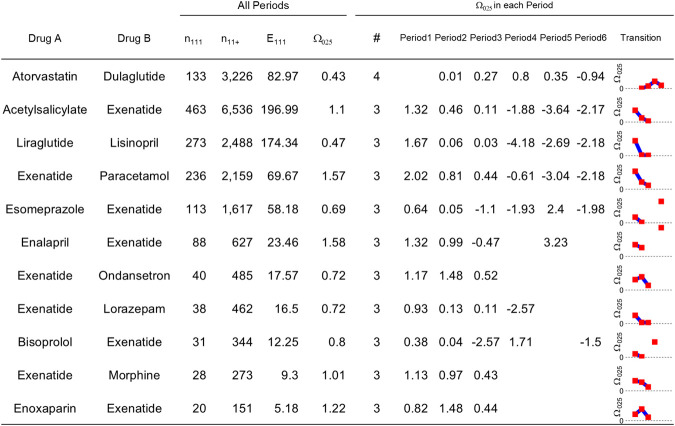
DDI analyses of AP between GLP-1 RAs or DPP-4 inhibitors and other medications (8,584 distinct agents) were performed, and the results obtained using the Ω shrinkage measure are presented for the entire study period as well as in period-stratified analyses. # represents the number of periods with positive signal; data are shown only when # > 2. The missing data reflect the absence of Ω_025_ values when either n_111_ or n_11+_ was zero.

To assess the temporal consistency of the interaction signals, we examined changes in the number of positive signals of Ω_025_ across predefined time periods. No drug pair demonstrated a consistently robust interaction signal across six or five periods. Among the evaluated combinations, the atorvastatin–dulaglutide pair showed positive interaction signals across four periods, representing the highest degree of temporal consistency observed in the analysis. Positive signals were identified for several drug pairs across three periods, including acetylsalicylate–exenatide, liraglutide–lisinopril, exenatide–paracetamol, esomeprazole–exenatide, enalapril–exenatide, exenatide–ondansetron, exenatide–lorazepam, bisoprolol–exenatide, exenatide–morphine, and enoxaparin–exenatide (Figure 4). In addition, 48 drug pairs exhibited positive interaction signals across two periods.

## 4 Discussion

Using FAERS, one of the world’s largest spontaneous reporting databases, we conducted disproportionality analyses of individual drugs associated with AP, as well as DDI analyses examining the pairwise (1:1) concomitant use of GLP-1 RAs or DPP-4 inhibitors with other medications. Several considerations are critical for the appropriate interpretation of the present findings. First, because this study was based on a spontaneous reporting database, causal relationships between drug exposure and AP could not be inferred. Second, our results should not be interpreted as quantitative risk estimates. Rather, the identified associations require external validation, and the primary role of this analysis is to support the prioritization of hypotheses for subsequent investigations. Accordingly, this study is exploratory in nature, providing an initial signal detection framework to guide further pharmacological and epidemiological evaluation.

In the disproportionality analyses, cRORs were elevated for GLP-1 RAs, DPP-4 inhibitors, and oral antidiabetic agents, such as metformin, indicating strong associations with reports of AP. A wide range of additional drugs also yielded high signal values, which were largely concordant with previously reported evidence (Li et al., 2024), thereby supporting the validity of the analysis.

GLP-1 RAs and DPP-4 inhibitors are incretin-based therapies that were first introduced with the approval of exenatide in 2005 and sitagliptin in 2006. Since their initial market entry, multiple agents within these classes have been approved and now play a central role in the management of type 2 diabetes mellitus. Liraglutide, originally approved for the treatment of type 2 diabetes in 2009, was the first GLP-1 RA approved globally for treating obesity in 2014. Given the expanding indications and increasing use of GLP-1 RAs, their clinical relevance is expected to increase. Although warnings regarding AP are included in product labels for both GLP-1 RAs and DPP-4 inhibitors, the causal relationship between these agents and AP remains a subject of ongoing debate (Zhang et al., 2022; Nreu et al., 2023; Saini et al., 2023; Pratley et al., 2024). Importantly, patients treated with these agents often have underlying conditions such as diabetes, obesity, or metabolic syndrome, each of which independently confers an elevated risk of pancreatitis, necessitating careful clinical interpretation that accounts for the baseline risk (Girman et al., 2010; Quan and Yang, 2024). In addition to antidiabetic medications, eluxadoline, a treatment for diarrhea-predominant irritable bowel syndrome, has also been implicated in AP development (Cash et al., 2021). Consistent with previous reports, eluxadoline showed a markedly elevated signal in this study. Additionally, drugs previously associated with AP—including acute lymphoblastic leukemia treatments, such as pegaspargase and asparaginase (Knoderer et al., 2007; M’harzi et al., 2022); antiretroviral agents, such as didanosine and stavudine (Reisler et al., 2005; Dragovic, 2013); and antipsychotics, such as quetiapine and olanzapine (He et al., 2022; Frey, 2025)—also showed elevated signals, further reflecting established clinical knowledge.

In the individual-drug disproportionality analyses, all drugs recorded in the reports were included regardless of their designated role (PS, SS, concomitant, or I). This inclusive approach was adopted because the primary objective of the present study was to evaluate DDI rather than quantify the contribution of individual suspected drugs alone. However, this strategy introduces potential indications and treatment bias, particularly through the inclusion of medications used for the management of pancreatitis. Accordingly, such drugs should be excluded from the interpretation to avoid distortion of signal estimates driven by reverse causality.

Regarding the pairwise DDI analysis with the Ω shrinkage measure model, in the pooled analysis covering the entire observation period, positive signals based on the Ω_025_ criterion were identified for 308 drug pairs. Drug combinations with the highest number of reports were insulin and exenatides. However, despite its high reporting frequency in the pooled dataset, this combination did not yield a positive shrinkage signal in any period-specific analysis. Notably, the pooled Ω_025_ value for this pair was 0.04, lying at the margin of the predefined signal-detection threshold. Insulin is widely used in the management of diabetes mellitus and may be administered to treat hyperglycemia secondary to AP. Therefore, the observed association may reflect confounding by indication or treatment-related bias rather than a true pharmacological interaction. The second most frequently reported combination was acetylsalicylate and exenatide, with 436 reports and an Ω_025_ value of 1.10. In this case, the number of reports for the observed co-occurrence (n_111_) exceeded the expected count (E_111_), resulting in a positive Ω_025_ signal.

Next, to assess temporal consistency, we examined the number of positive signals observed across individual reporting periods. Across the six periods, no drug combination exhibited positive signals in five or more periods, and no combination demonstrated interaction signals that were consistently observed across all periods. Eleven drug pairs showed positive signals in four or three periods, all of which involved GLP-1 RAs. Among these, the atorvastatin–dulaglutide combination showed the highest temporal recurrence, with positive signals observed consecutively from Periods 2 to 5. Drug pairs with positive signals in two periods totaled 48, of which 46 pairs involved GLP-1 RAs, and only two pairs involved DPP-4 inhibitors. Among these, most drug pairs involved exenatides.

Exenatide was the first GLP-1 RA to receive regulatory approval and was subsequently accompanied by an accumulation of post-marketing reports on AP, which initiated broader debate regarding the potential association between GLP-1 RAs and AP. Therefore, the predominance of positive signals observed during the earlier reporting periods may reflect this historical context, including heightened awareness following market introduction and the possibility of transient stimulated reporting. Accordingly, the potential influence of temporal reporting dynamics should be considered when interpreting these findings.

Among the drug pairs that exhibited positive signals in three or four reporting periods, combinations of statins and angiotensin-converting enzyme (ACE) inhibitors were identified. These drug classes have previously been reported to be associated with AP (Maringhini et al., 1997; Carnovale et al., 2003; Kanbay et al., 2006; Deshpande et al., 2011; Syed et al., 2021; Masarweh et al., 2023; Alshammari et al., 2025), and the present disproportionality analysis likewise demonstrated significantly elevated cRORs for their individual use. This observation prompted further investigation of potential DDI signals between GLP-1 RAs and statins or ACE inhibitors, as detailed in the Supplementary Materials S2, S3. However, no consistent pattern of increased reporting was observed for these drug combinations, and no sustained tendency toward enhanced reporting emerged across the reporting periods. Among other combinations, the exenatide–ondansetron and exenatide–paracetamol (acetaminophen) pairs exhibited positive signals in three reporting periods. Although such associations are infrequent, prior reports have described links between paracetamol and AP (He et al., 2018), as well as between ondansetron and AP (Alberti-Flor, 1995), which may have contributed, in part, to the observed signals, warranting further investigation.

Analyses based on the entire observation period, and those stratified by discrete time intervals, have distinct methodological strengths and limitations. Pooled analysis across the entire observation period offers the advantage of increased statistical power owing to larger sample sizes, thereby enhancing the sensitivity of signal detection. However, this approach does not allow for the assessment of the temporal consistency of detected signals. As a result, signals identified in the pooled analyses may reflect transient reporting phenomena rather than stable drug–event associations. By contrast, period-specific analyses enable the identification of signals that might be obscured when the data are averaged across the entire study period. This approach facilitates the evaluation of signal consistency over time and may help mitigate the impact of stimulated reporting associated with regulatory actions, media attention, or changes in clinical practice. A period-stratified approach may facilitate the prioritization of candidate signals by assessing temporal consistency across multiple signals emerging from pooled analyses. However, stratification by time inevitably reduces the number of reports within each period, leading to diminished statistical power and an increased risk of false-negative findings. Given these complementary strengths and weaknesses, pooled and period-specific analyses should not be viewed as competing approaches, but rather as mutually reinforcing strategies. Their combined application allows for a more nuanced and robust interpretation of pharmacovigilance signals, balancing sensitivity with temporal validity.

Several limitations inherent to studies based on spontaneous reporting databases should be acknowledged. First, issues of under-reporting and over-reporting are unavoidable, and causal relationships between drug exposure and adverse events cannot be established from such data. Second, the present analysis was restricted to pairwise DDIs, and the potential effects of concomitant use of three or more drugs were not evaluated. In addition, relevant clinical and demographic factors, including age, sex, underlying comorbidities (such as hepatic or renal dysfunction), dosage information, formulation, dosing frequency, route of administration, and lifestyle factors (e.g., alcohol consumption), were not accounted for in the analysis. Concomitant drug use was defined as medications listed within the same report. Consequently, detailed information on treatment duration, temporal sequencing, or the exact overlap of drug exposure was unavailable, and some reports may have included medications administered before or after treatment switches, rather than truly concurrent use. In real-world clinical practice, interpretation of drug safety signals requires careful consideration of patient-specific contextual factors. Regarding the temporal analysis, drug availability may vary across time periods. Recently approved drugs are not captured in earlier periods, whereas some older drugs may be reported less frequently due to the introduction of newer agents or changes in therapeutic guidelines. This may be reflected in Figure 4, where some missing values of period-specific Ω_025_ were observed for exenatide combinations, particularly in later periods. Additionally, dividing the data into a larger number of time periods may result in an insufficient number of cases to ensure adequate statistical power, whereas using broader time intervals may limit the ability to assess the robustness of the observed signals. Therefore, the optimal division of time periods remains uncertain and should be considered a limitation of this study. Given these limitations, the findings of this study should be interpreted as hypothesis-generating and do not necessarily reflect the actual clinical risk. Verification of these hypotheses requires further investigation, including pharmacological mechanistic studies and epidemiological validation using other large-scale healthcare databases. Despite these limitations, a comprehensive analysis of the world’s largest spontaneous reporting data source remains a valuable initial step toward risk assessment. Such large-scale approaches are particularly well-suited for detecting subtle safety signals that are difficult to identify at the level of individual case reports, thereby providing an important foundation for subsequent confirmatory research.

## 5 Conclusion

Our analyses demonstrated that period-stratified analysis effectively reduced false-positive signals identified in the pooled DDI analyses. No concomitant medications were found to clearly increase AP reporting when combined with GLP-1 RA or DPP-4 inhibitors. Nevertheless, specific drug pairs exhibited weak but recurrent signals across multiple reporting periods, including dulaglutide–atorvastatin, suggesting that these combinations warrant continued pharmacovigilance, cautious interpretation, and longitudinal monitoring during post-marketing safety surveillance.

## Data Availability

Publicly available datasets were analyzed in this study. This data can be found here: https://fis.fda.gov/extensions/FPD-QDE-FAERS/FPD-QDE-FAERS.html.
