# Supplementary material for: Comprehensive pharmacovigilance assessment of pairwise drug–drug interactions and acute pancreatitis in the FDA adverse event reporting system: focus on incretin-based drugs
Source: Front Drug Saf Regul. 2026 Jun 10;6:1827098. doi: 10.3389/fdsfr.2026.1827098 (PMC13291152; doi:10.3389/fdsfr.2026.1827098)
Supplement: Supplementary file 1 [file Presentation1.pptx]

## Slide 1
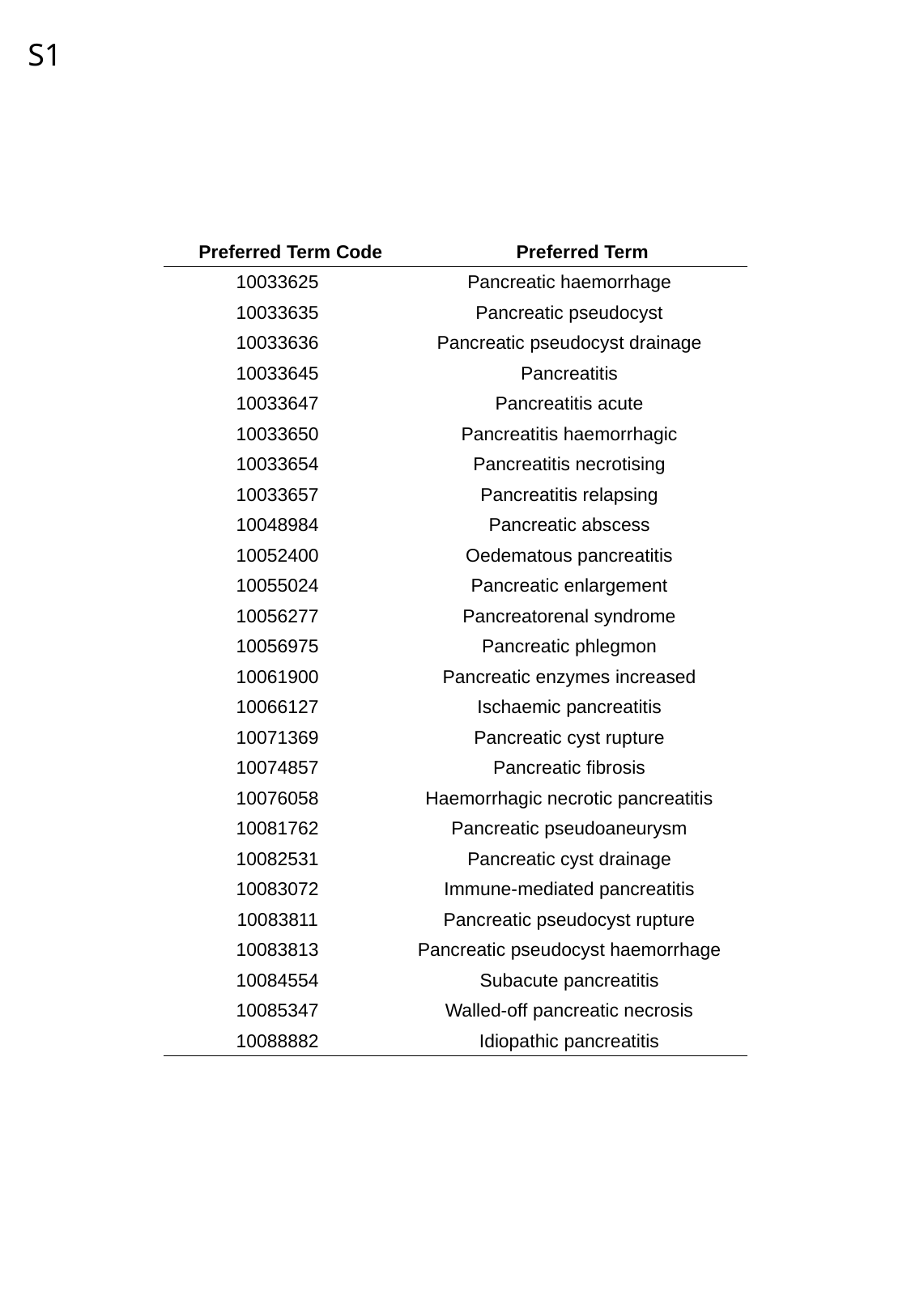

S1
| Preferred Term Code | Preferred Term |
| --- | --- |
| 10033625 | Pancreatic haemorrhage |
| 10033635 | Pancreatic pseudocyst |
| 10033636 | Pancreatic pseudocyst drainage |
| 10033645 | Pancreatitis |
| 10033647 | Pancreatitis acute |
| 10033650 | Pancreatitis haemorrhagic |
| 10033654 | Pancreatitis necrotising |
| 10033657 | Pancreatitis relapsing |
| 10048984 | Pancreatic abscess |
| 10052400 | Oedematous pancreatitis |
| 10055024 | Pancreatic enlargement |
| 10056277 | Pancreatorenal syndrome |
| 10056975 | Pancreatic phlegmon |
| 10061900 | Pancreatic enzymes increased |
| 10066127 | Ischaemic pancreatitis |
| 10071369 | Pancreatic cyst rupture |
| 10074857 | Pancreatic fibrosis |
| 10076058 | Haemorrhagic necrotic pancreatitis |
| 10081762 | Pancreatic pseudoaneurysm |
| 10082531 | Pancreatic cyst drainage |
| 10083072 | Immune-mediated pancreatitis |
| 10083811 | Pancreatic pseudocyst rupture |
| 10083813 | Pancreatic pseudocyst haemorrhage |
| 10084554 | Subacute pancreatitis |
| 10085347 | Walled-off pancreatic necrosis |
| 10088882 | Idiopathic pancreatitis |

## Slide 2
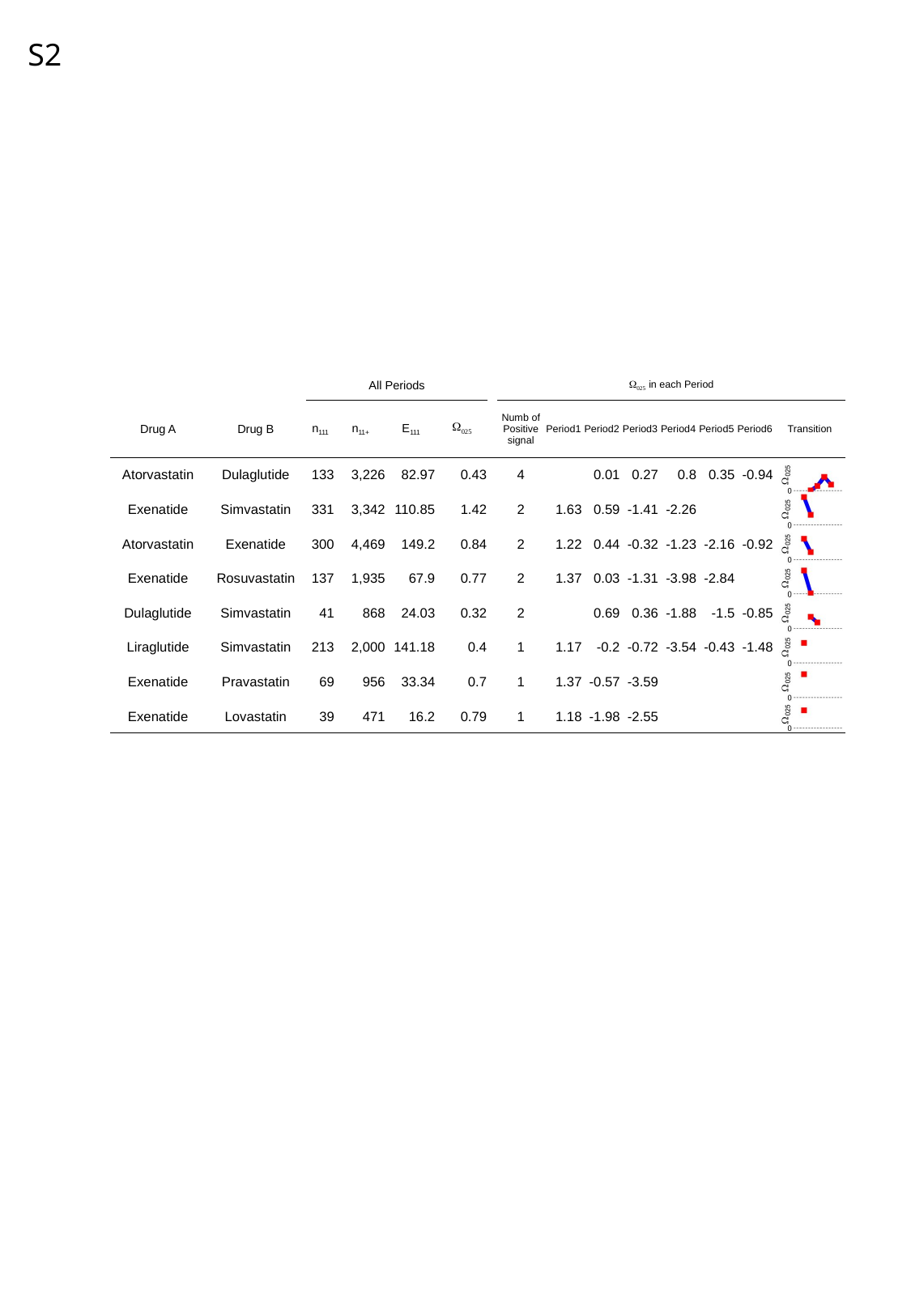

S2
| | | All Periods | | | | | W025 in each Period | | | | | | | |
| --- | --- | --- | --- | --- | --- | --- | --- | --- | --- | --- | --- | --- | --- | --- |
| Drug A | Drug B | n111 | n11+ | E111 | W025 | | Numb of Positive signal | Period1 | Period2 | Period3 | Period4 | Period5 | Period6 | Transition |
| Atorvastatin | Dulaglutide | 133 | 3,226 | 82.97 | 0.43 | | 4 | | 0.01 | 0.27 | 0.8 | 0.35 | -0.94 | |
| Exenatide | Simvastatin | 331 | 3,342 | 110.85 | 1.42 | | 2 | 1.63 | 0.59 | -1.41 | -2.26 | | | |
| Atorvastatin | Exenatide | 300 | 4,469 | 149.2 | 0.84 | | 2 | 1.22 | 0.44 | -0.32 | -1.23 | -2.16 | -0.92 | |
| Exenatide | Rosuvastatin | 137 | 1,935 | 67.9 | 0.77 | | 2 | 1.37 | 0.03 | -1.31 | -3.98 | -2.84 | | |
| Dulaglutide | Simvastatin | 41 | 868 | 24.03 | 0.32 | | 2 | | 0.69 | 0.36 | -1.88 | -1.5 | -0.85 | |
| Liraglutide | Simvastatin | 213 | 2,000 | 141.18 | 0.4 | | 1 | 1.17 | -0.2 | -0.72 | -3.54 | -0.43 | -1.48 | |
| Exenatide | Pravastatin | 69 | 956 | 33.34 | 0.7 | | 1 | 1.37 | -0.57 | -3.59 | | | | |
| Exenatide | Lovastatin | 39 | 471 | 16.2 | 0.79 | | 1 | 1.18 | -1.98 | -2.55 | | | | |

## Slide 3
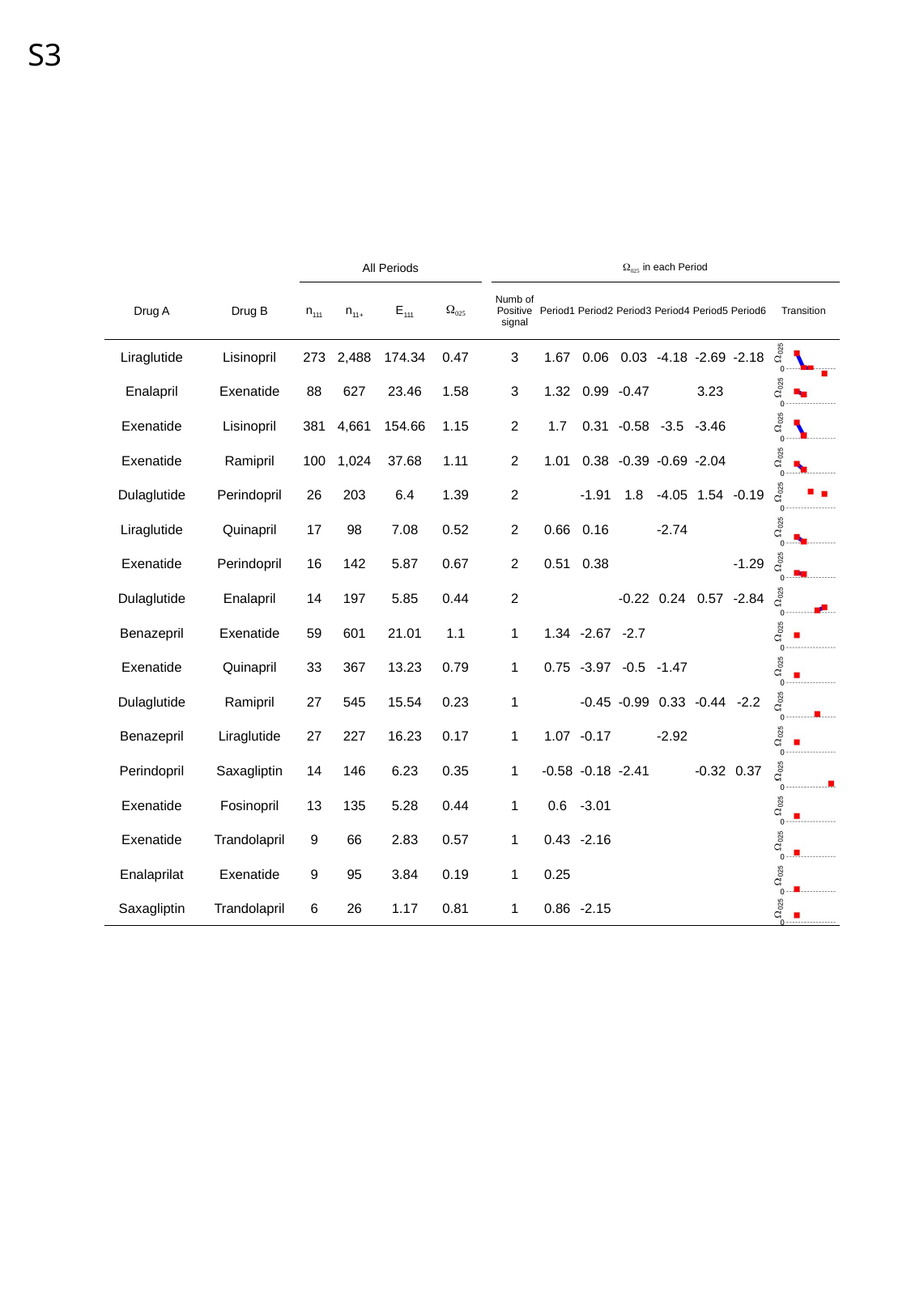

S3
| | | All Periods | | | | | W025 in each Period | | | | | | | |
| --- | --- | --- | --- | --- | --- | --- | --- | --- | --- | --- | --- | --- | --- | --- |
| Drug A | Drug B | n111 | n11+ | E111 | W025 | | Numb of Positive signal | Period1 | Period2 | Period3 | Period4 | Period5 | Period6 | Transition |
| Liraglutide | Lisinopril | 273 | 2,488 | 174.34 | 0.47 | | 3 | 1.67 | 0.06 | 0.03 | -4.18 | -2.69 | -2.18 | |
| Enalapril | Exenatide | 88 | 627 | 23.46 | 1.58 | | 3 | 1.32 | 0.99 | -0.47 | | 3.23 | | |
| Exenatide | Lisinopril | 381 | 4,661 | 154.66 | 1.15 | | 2 | 1.7 | 0.31 | -0.58 | -3.5 | -3.46 | | |
| Exenatide | Ramipril | 100 | 1,024 | 37.68 | 1.11 | | 2 | 1.01 | 0.38 | -0.39 | -0.69 | -2.04 | | |
| Dulaglutide | Perindopril | 26 | 203 | 6.4 | 1.39 | | 2 | | -1.91 | 1.8 | -4.05 | 1.54 | -0.19 | |
| Liraglutide | Quinapril | 17 | 98 | 7.08 | 0.52 | | 2 | 0.66 | 0.16 | | -2.74 | | | |
| Exenatide | Perindopril | 16 | 142 | 5.87 | 0.67 | | 2 | 0.51 | 0.38 | | | | -1.29 | |
| Dulaglutide | Enalapril | 14 | 197 | 5.85 | 0.44 | | 2 | | | -0.22 | 0.24 | 0.57 | -2.84 | |
| Benazepril | Exenatide | 59 | 601 | 21.01 | 1.1 | | 1 | 1.34 | -2.67 | -2.7 | | | | |
| Exenatide | Quinapril | 33 | 367 | 13.23 | 0.79 | | 1 | 0.75 | -3.97 | -0.5 | -1.47 | | | |
| Dulaglutide | Ramipril | 27 | 545 | 15.54 | 0.23 | | 1 | | -0.45 | -0.99 | 0.33 | -0.44 | -2.2 | |
| Benazepril | Liraglutide | 27 | 227 | 16.23 | 0.17 | | 1 | 1.07 | -0.17 | | -2.92 | | | |
| Perindopril | Saxagliptin | 14 | 146 | 6.23 | 0.35 | | 1 | -0.58 | -0.18 | -2.41 | | -0.32 | 0.37 | |
| Exenatide | Fosinopril | 13 | 135 | 5.28 | 0.44 | | 1 | 0.6 | -3.01 | | | | | |
| Exenatide | Trandolapril | 9 | 66 | 2.83 | 0.57 | | 1 | 0.43 | -2.16 | | | | | |
| Enalaprilat | Exenatide | 9 | 95 | 3.84 | 0.19 | | 1 | 0.25 | | | | | | |
| Saxagliptin | Trandolapril | 6 | 26 | 1.17 | 0.81 | | 1 | 0.86 | -2.15 | | | | | |
